# Effects of Urolithin A supplementation on muscle health outcomes in humans from randomized controlled trials

**DOI:** 10.3389/fnut.2026.1834344

**Published:** 2026-07-22

**Authors:** Tam Dao, Thanh T. Nguyen, Karim Gariani, Hyun Jin Kim, Dongryeol Ryu

**Affiliations:** 1 Department of Physiology, Sungkyunkwan University School of Medicine, Suwon, Republic of Korea; 2 Department of Biomedical Science and Engineering, Gwangju Institute of Science and Technology, Gwangju, Republic of Korea; 3 Service of Endocrinology, Diabetes and Metabolism, Geneva University Hospitals, Genève, Switzerland; 4 Faculty of Medicine, University of Geneva, Genève, Switzerland

**Keywords:** clinical trial, Meta-analysis, physical functional performance, skeletal muscle, systematic review, Urolithin A, walk test

## Abstract

**Background:**

Urolithin A (UA), a gut microbiota–derived metabolite, has been proposed to improve skeletal muscle function, but evidence from randomized controlled trials has not been systematically summarized. This systematic review and meta-analysis aimed to evaluate the effects of UA supplementation on muscle performance as well as biochemical and mitochondrial biomarkers in humans.

**Methods:**

Randomized controlled trials published up to December 2025 were identified through systematic searches of four electronic databases: PubMed, Embase, Web of Science, and Scopus. Trials comparing oral UA supplementation with placebo and reporting muscle-related outcomes were included. Risk of bias was assessed using the Cochrane Risk of Bias 2 (RoB 2) tool, and the certainty of evidence for the primary quantitatively synthesized outcome was rated using the GRADE approach. Quantitative meta-analysis was feasible only for the 6-min walk test (6MWT); remaining outcomes were synthesized narratively. For multi-arm trials with a shared comparator, intervention arms were combined into a single group following Cochrane Handbook guidance to avoid unit-of-analysis errors. Pooled mean differences with 95% confidence intervals were calculated using inverse-variance weighting, with both fixed-effect and random-effects sensitivity analyses.

**Results:**

Five randomized controlled trials (*n* = 236) were included. Quantitative synthesis was feasible only for the 6MWT (*k* = 2). After combining the 500 mg and 1,000 mg Urolithin A arms of Singh et al. (5) against the shared placebo group, the pooled mean difference was +17.03 m (95% CI −5.33 to 39.40 m; *p* = 0.135; *I*^2^ = 0%); a sensitivity analysis using placebo-group splitting yielded a comparable estimate (+18.80 m; 95% CI −3.24 to 40.85 m; *p* = 0.095). The certainty of evidence for the 6MWT outcome was rated as low (GRADE). Non-6MWT outcomes — muscle strength, endurance, aerobic capacity, and biochemical or mitochondrial biomarkers — were heterogeneous across populations, doses, and assessment modalities, were not quantitatively pooled, and are reported as exploratory signals rather than reproducible effects.

**Conclusion:**

The currently available randomized human evidence is limited to five small, short-term trials in clinically heterogeneous populations. Quantitative pooling was feasible for a single outcome (6MWT) based on two trials and showed a directionally favorable but statistically inconclusive effect with low GRADE certainty. Narrative findings on strength, endurance, and mitochondrial-related biomarkers are exploratory and hypothesis-generating, not reproducible evidence of efficacy. Larger, longer, and methodologically standardized trials in better-defined populations are required before firm clinical recommendations can be made.

**Systematic review registration:**

https://www.crd.york.ac.uk/PROSPERO/view/CRD420251270987, identifier: PROSPERO 2025 CRD420251270987.

## 1 Introduction

Age-associated declines in skeletal muscle mass and function (sarcopenia) lead to mobility limitations, loss of independence, and adverse health outcomes. Mitochondrial dysfunction and impaired mitochondrial quality control, particularly reduced mitophagy, are prominent features of aging muscle and are implicated in diminished bioenergetic capacity and performance. Urolithin A (UA) is a gut microbiota–derived postbiotic metabolite of dietary ellagitannins that has emerged as a candidate therapy to enhance mitochondrial quality control and muscle function by activating mitophagy pathways (PINK1–Parkin) and improving mitochondrial efficiency (1, 2).

Mechanistic and preclinical evidence support UA’s biological plausibility for muscle health. In *Caenorhabditis elegans*, UA induces mitophagy, preserves mitochondrial respiratory capacity, prolongs mobility, and extends lifespan; in rodents, oral UA improves exercise capacity and muscle function, consistent with enhanced mitochondrial quality control (1). Across cellular and animal models, UA increases canonical mitophagy/autophagy markers (e.g., LC3-II/LC3-I, PINK1, Parkin) and decreases p62, with additional engagement of receptor-mediated mitophagy (BNIP3/NIX) and SIRT1–PGC-1α signaling; functional gains include improvements in endurance, grip strength, and tetanic force and restoration of mitochondrial respiration and ATP levels (2, 3). These molecular signatures provide translational biomarkers for human studies.

Human trials have progressed from safety and pharmacodynamic studies to randomized controlled trials (RCTs) assessing functional endpoints. In a randomized, placebo-controlled, multiple-ascending-dose study in older adults, UA (250–1,000 mg/day for 28 days; single doses up to 2,000 mg) was safe and induced a molecular signature consistent with improved mitochondrial and cellular health, with detectable exposure in skeletal muscle (4). In the ATLAS RCT of untrained, overweight, middle-aged adults, 4 months of UA (500 or 1,000 mg/day) improved leg muscle strength (~10–12%) and measures of aerobic endurance, and favorably modulated plasma acylcarnitines and skeletal-muscle mitophagy proteins, without changes in body composition, supporting efficacy on muscle performance with mitochondrial biomarker concordance (5). In resistance-trained male athletes, 8 weeks of UA 1,000 mg/day improved maximum voluntary isometric contraction and repetitions to failure, with mixed effects on inflammatory and oxidative stress markers, and no major safety concerns (6).

UA’s origin as a microbiome-derived metabolite highlights interindividual variability in endogenous production (urolithin metabotypes), motivating direct supplementation to achieve consistent systemic exposure and suggesting potential heterogeneity of response across populations. Reviews emphasize variability in UM-A/UM-B/UM-0 metabotypes and the rationale for exogenous UA to bypass poor endogenous conversion, while underscoring UA’s safety profile and mitophagy-targeted mechanism distinct from broader autophagy inducers (3). Together, these data support clinical rationale to synthesize RCT evidence on UA’s effects on strength, endurance, physical performance, and mitochondrial biomarkers, and to systematically evaluate dosing, duration, safety, and moderators such as age, training status, and microbiome-related variability (2, 4, 5).

Despite these encouraging findings, the available evidence base remains limited. The number of randomized controlled trials is small, sample sizes are modest, intervention durations are short, and the included populations are clinically heterogeneous, spanning older adults, untrained middle-aged individuals, and trained or late-adolescent athletic cohorts. These features substantially constrain the feasibility of quantitative pooling and the strength of conclusions that can currently be drawn.

Given the growing interest in Urolithin A for muscle health and its potential relevance to conditions characterized by mitochondrial dysfunction and functional decline, a structured synthesis of the existing randomized human evidence is warranted. The present systematic review and meta-analysis was conducted with a PICOS-defined eligibility framework in adults and post-pubertal athletic populations. The aims were to (i) provide a PRISMA 2020-compliant narrative synthesis of muscle strength, endurance, physical performance, and mitochondrial or metabolic biomarker outcomes following oral Urolithin A supplementation; (ii) where feasible, quantitatively pool effect estimates; (iii) appraise the risk of bias of included trials using the Cochrane RoB 2 tool and the certainty of evidence for the primary pooled outcome using GRADE; and (iv) identify methodological limitations and priorities for future trials (7). We did not pre-specify broad clinical claims regarding aging-related functional decline, given the limited number and heterogeneity of available studies.

By doing so, this review will provide a consolidated evidence base for UA supplementation in muscle health, and may inform future clinical trial design, dosage optimization, and potential clinical or nutritional applications.

## 2 Methods

### 2.1 Registration and reporting framework

This systematic review and meta-analysis was prospectively registered with PROSPERO (ID CRD420251270987) and was conducted in accordance with the Preferred Reporting Items for Systematic Reviews and Meta-Analyses (PRISMA) 2020 statement (8).

### 2.2 Literature search strategy

A comprehensive literature search was performed across four electronic databases — PubMed, Embase, Web of Science, and Scopus — covering all records available up to 15 December 2025 (final search date). The search was limited to studies published in English. The full Boolean search strategy, adapted as appropriate for each database, is presented below and is also provided per-database in Supplementary Table S1. Reference lists of included studies and relevant reviews were manually screened to identify additional eligible trials.

### 2.3 Eligibility criteria

Eligibility criteria were defined *a priori* using the PICOS framework, summarized in Supplementary Table S2 (PICOS table).

Inclusion of one trial in academy soccer players (mean age ~17.5 years) was retained because participants were post-pubertal competitive athletes with physiological characteristics comparable to young adult athletic populations; this is acknowledged as a source of clinical heterogeneity in the Discussion.

Studies were excluded if they were non-randomized, non-human, review articles, conference abstracts, editorials, or lacked extractable outcome data.

**Figure 1:**
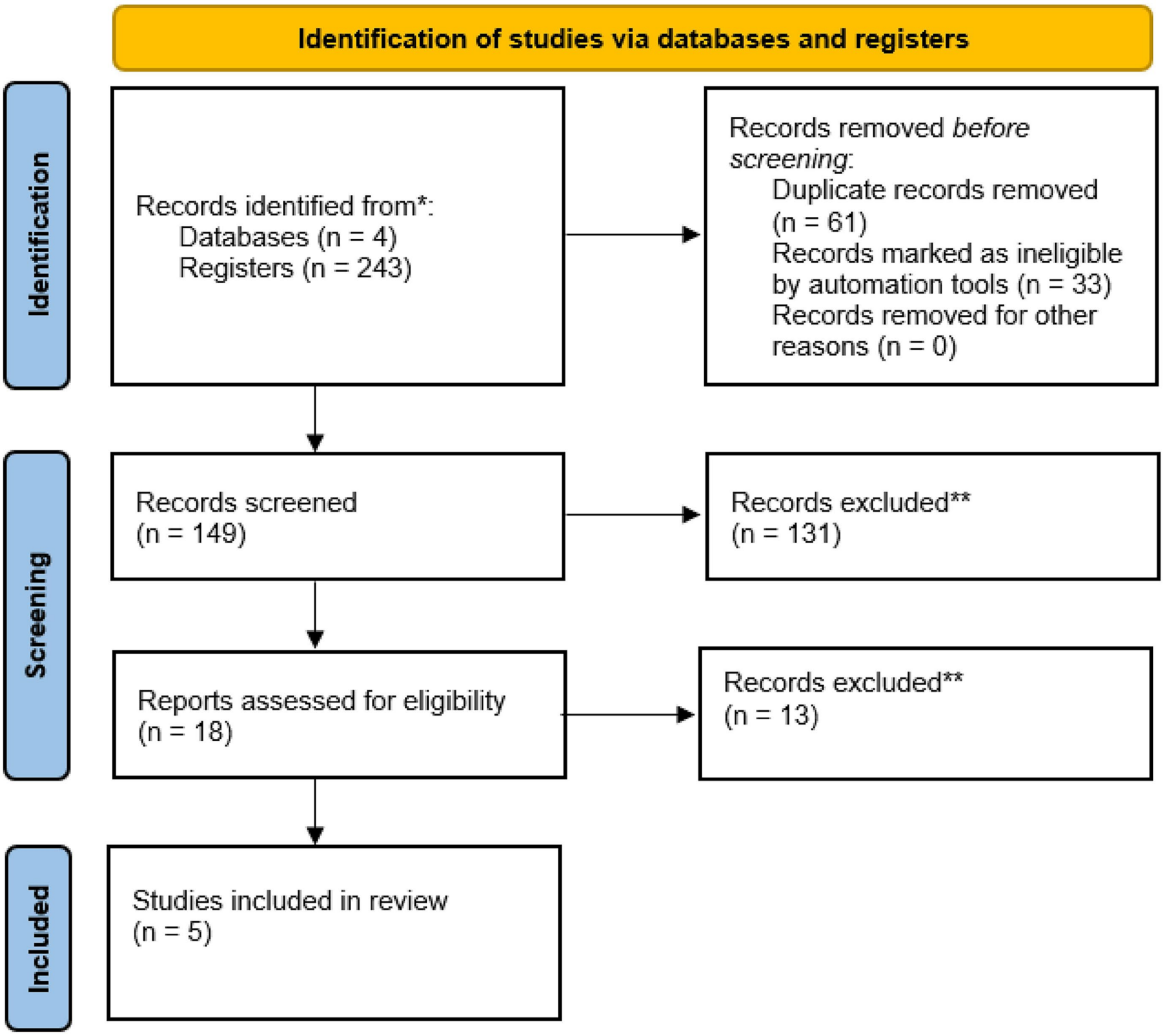
PRISMA 2020 flow diagram for the selection of studies included in the systematic review.

All retrieved records were imported into Covidence (Veritas Health Innovation, Melbourne, Australia) for de-duplication, screening, and data management. Covidence’s automated duplicate-detection function was used during the import process, resulting in the removal of 61 duplicate records. In addition, 33 records were automatically flagged as ineligible based on duplicate or bibliographic matching criteria and were subsequently manually reviewed and confirmed by the investigators before exclusion. Two reviewers independently screened titles and abstracts against the eligibility criteria, followed by independent full-text assessment of potentially relevant articles. Discrepancies at either stage were resolved through discussion until consensus was reached, with a third reviewer consulted when necessary. The study selection process is presented in the PRISMA 2020 flow diagram (Figure 1).

### 2.4 Data extraction

For outcomes not directly reported as between-group comparisons, change-from-baseline values were calculated using arm-level summary statistics in accordance with Cochrane Handbook §6.5 guidance. For Singh et al. (5), Day 120 intention-to-treat change-from-baseline values for the placebo, 500 mg, and 1,000 mg Urolithin A arms (placebo: *n* = 27, −0.52 ± 59.08 m; 500 mg: *n* = 27, −0.74 ± 76.10 m; 1,000 mg: *n* = 25, +33.43 ± 51.07 m) were confirmed by the corresponding author and used in the revised meta-analysis; no correlation-coefficient assumption or imputation of change-score SD was required, since author-confirmed change-score SDs were directly available. For Liu et al. (9), published change-from-baseline means and SDs were used as reported. Between-group mean differences were calculated as UA minus placebo, with the corresponding standard error derived from arm-level SDs and sample sizes.

Handling of multi-arm trials. Singh et al. (5) compared two Urolithin A arms (500 mg/day and 1,000 mg/day) against a single shared placebo group. Inclusion of only the 1,000 mg arm in the previous analysis was not pre-specified and would introduce a unit-of-analysis error (Cochrane Handbook §6.5.2.10, §23.3.4). In the revised meta-analysis, the two Urolithin A arms were therefore combined into a single intervention group against the shared placebo, using the standard Cochrane formulae for pooling means and standard deviations:

n_UA = 25 + 27 = 52 M_UA = (25·33.43 + 27·(−0.74))/52 = +15.69 m SD_UA = √[((24·51.07^2^ + 26·76.10^2^) + (25·27/52)·(33.43 – (−0.74))^2^)/51] = 66.91 m

A sensitivity analysis using placebo-group splitting (Cochrane Option B) was also performed and produced comparable results.

Where direct change-score SDs were not reported, they were derived from arm-level summary statistics in accordance with Cochrane Handbook guidance. No imputation using assumed pre–post correlation coefficients was required for the studies included in the 6MWT meta-analysis. All effect sizes were expressed as mean differences (MDs) with 95% confidence intervals.

### 2.5 Bias assessment

The methodological quality of each included randomized controlled trial was independently evaluated by two reviewers using the Cochrane Risk of Bias 2 (RoB 2) tool (10). The following domains were assessed: (1) bias arising from the randomization process; (2) bias due to deviations from intended interventions; (3) bias due to missing outcome data; (4) bias in measurement of the outcome; and (5) bias in selection of the reported result. Each domain was rated as “low risk,” “some concerns,” or “high risk” according to the Cochrane Handbook criteria. Disagreements were resolved through discussion to reach consensus.

### 2.6 Statistical analysis

Meta-analyses were performed using the metafor package (R software). For the 6MWT, between-study pooled mean differences were estimated using inverse-variance weighting. Because only two studies contributed to the pooled estimate (*k* = 2), heterogeneity statistics (Cochran’s Q, *I*^2^) are recognized to be underpowered and were interpreted with caution. Both a fixed-effect (inverse-variance) and a random-effects (DerSimonian–Laird) model were fitted as pre-specified sensitivity analyses; the random-effects estimate of between-study variance (τ^2^) was 0, so fixed-effect and random-effects estimates were identical. A further sensitivity analysis using the placebo-splitting approach for the multi-arm Singh trial was also conducted. The certainty of evidence for the 6MWT outcome was rated using the GRADE approach across risk of bias, inconsistency, indirectness, imprecision, and publication bias domains, and a Summary of Findings table is provided in the Supplementary materials. Outcomes other than the 6MWT were summarized narratively because of the small number of trials, varying outcome definitions, and methodological heterogeneity, which precluded meaningful quantitative pooling. Two-sided *p* < 0.05 was used as the threshold for statistical significance, with effect estimates and confidence intervals emphasized over dichotomous interpretations.

## 3 Results

### 3.1 Study selection

The database search identified 243 records across the four databases (PubMed, Embase, Web of Science, and Scopus). After removal of 61 duplicate records identified by Covidence’s automated duplicate-detection function and verified manually, and exclusion of 33 records flagged as ineligible by automated tool and confirmed by manual review, 149 records remained for title and abstract screening. Of these, 131 were excluded for not meeting the eligibility criteria.

Eighteen full-text articles were retrieved and assessed for eligibility. Of these, 13 studies were excluded due to reasons such as non-randomized design, absence of relevant outcomes, or duplicate data. Ultimately, five randomized controlled trials met the inclusion criteria and were included in the systematic review (Figure 1).

### 3.2 Study characteristics

Five randomized controlled trials met the inclusion criteria, comprising a total of 236 participants across diverse populations ranging from older adults to trained athletes. The duration of UA supplementation varied from 4 weeks to 4 months, and daily doses ranged from 500 mg to 1,000 mg administered orally.

Baseline demographic and physiological characteristics of participants from each randomized controlled trial are presented in Supplementary Table S3. Across all studies, participant characteristics were well balanced between the UA and placebo groups, confirming adequate randomization.

Two trials [Singh et al., (5); Liu et al., (9)] were conducted in middle-aged or older adults, primarily targeting mitochondrial and functional muscle outcomes such as hamstring strength, endurance, and 6-min walk distance (5, 9). The remaining trials [Zhao et al., (6); Whitfield et al. (11); Acevedo et al., (12)] enrolled physically active or trained male participants, including resistance-trained athletes, endurance runners, and competitive soccer players (6, 11, 12). These studies predominantly focused on performance-related outcomes such as maximal voluntary contraction, repetition-to-failure endurance, aerobic capacity (VO₂max or Yo-Yo intermittent recovery performance), neuromuscular power, and exercise-induced biochemical markers.

Across studies, outcome assessments included functional performance tests, muscle strength and endurance measures, and biochemical biomarkers indicative of mitochondrial function, oxidative stress, and inflammation. All studies used randomized, placebo-controlled designs; however, populations, intervention durations, and outcome assessments were heterogeneous, and quantitative pooling proved feasible only for the 6MWT (Table 1).

**Table 1:** Characteristics of included randomized controlled trials investigating Urolithin A supplementation and muscle-related outcomes.

Study (first author, year)	Population (n; age; sex)	Intervention (dose; duration)	Comparator	Primary/relevant outcomes
Liu et al., 2022 (9) (*JAMA Network Open*)	66 older adults (mean 71.7 yrs.; 33 UA / 33 placebo; ~75.8% female)	UA 1000 mg/day; 4 months	Placebo	6-min walk distance; maximal ATP production; muscle endurance; plasma biomarkers (acylcarnitines, ceramides)
Singh et al., 2022 (5) (*Cell Reports Medicine*)	88 middle-aged overweight untrained adults	UA 500 mg or 1,000 mg/day; 4 months	Placebo	Muscle strength (hamstring, knee flexion, hand-grip); aerobic endurance (VO₂peak); biomarkers (acylcarnitines, CRP)
Zhao et al., 2024 (6) (*J. Int. Soc. Sports Nutr.*)	20 resistance-trained male athletes (mean 24 yrs.; training ~4.4 yrs)	UA 1 g/day; 8 weeks	Placebo	Muscle strength (1RM bench press, squat), max voluntary isometric contraction; endurance (reps to failure); oxidative stress and inflammatory markers
Whitfield et al., 2025 (11) (*Sports Medicine*)	42 competitive male distance runners (mean 27.2 yrs.; VO₂max ≈ 66.4 mL·kg^−1^·min^−1^)	UA 1000 mg/day; 4 weeks (at altitude 1,700–2,200 m)	Placebo	Body composition; hemoglobin mass; running economy; VO₂max; CRP; creatine kinase
Monsalve Acevedo et al., 2025 (12) (Front Nutr)	20 male academy soccer players (mean ~17.5 yrs.; UA n = 10 / placebo n = 10)	UA 1000 mg/day; 6 weeks (preseason training)	Placebo	Aerobic endurance; lower-limb strength/power; maximal sprint speed; salivary antioxidant capacity

### 3.3 Risk of bias assessment

**Figure 2:**
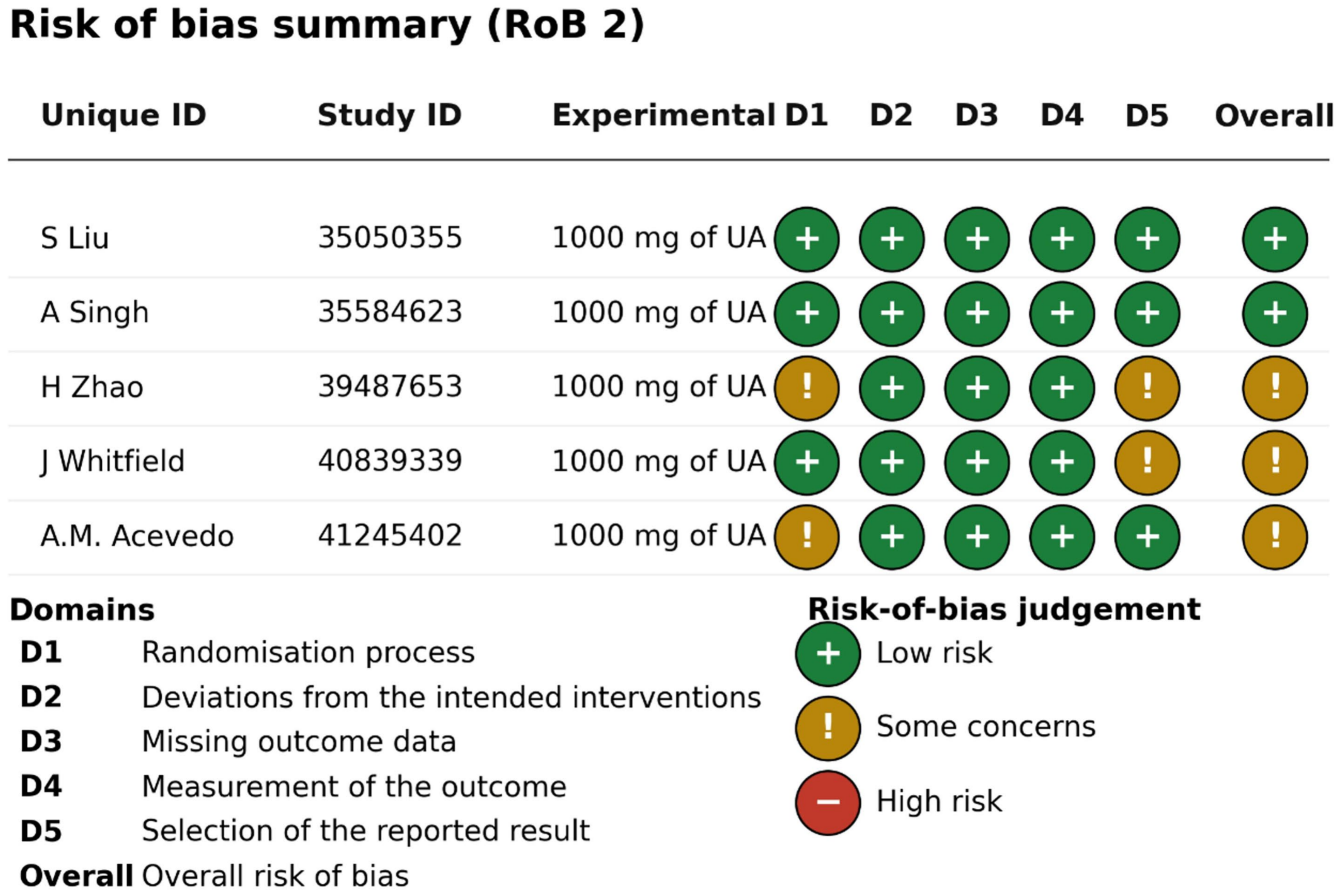
Summary of risk of bias assessment across included randomized controlled trials using the Cochrane RoB 2 tool.

The methodological quality of the five randomized controlled trials was assessed using the Cochrane Risk of Bias 2 (RoB 2) tool (Figure 2). Overall, two studies [Liu et al., (9); Singh et al., (5)] were judged to have a low risk of bias, while the remaining three studies [Zhao et al., (6); Whitfield et al. (11); Acevedo et al., (12)] were rated as having some concerns. No study was classified as having a high risk of bias.

Across domains, deviations from intended interventions (D2), missing outcome data (D3), and measurement of the outcome (D4) were consistently judged as low risk in all included trials. Some concerns related to the randomization process (D1) were identified in Zhao et al. (6) and Acevedo et al. (12), primarily due to insufficient reporting of random sequence generation or allocation concealment procedures. In addition, some concerns in the selection of the reported result (D5) were observed in Zhao et al. (6) and Whitfield et al. (11), reflecting the absence of clearly prespecified analysis plans or trial registration information. Despite these issues, no study demonstrated a pattern of bias severe enough to warrant an overall high-risk judgment.

### 3.4 Muscle strength

Across the five included randomized controlled trials, muscle strength was assessed using heterogeneous protocols, including hamstring and quadriceps torque, maximal voluntary isometric contraction, hand-grip strength, and one-repetition maximum bench-press and squat. Numerical changes more often favored Urolithin A than placebo, but only isolated outcomes reached statistical significance in the original trials [e.g., hamstring average peak torque in Singh et al., (5), +19.6%; quadriceps MVIC in Zhao et al., (6)]. Bench-press and squat 1RM outcomes in Zhao et al. (6) were not significantly different from placebo (*p* = 0.46 and *p* = 0.71, respectively); the significance markers previously shown in Table 2 for these outcomes were incorrect and have been corrected. Because of heterogeneous outcome definitions, differing units, and small sample sizes, these strength findings were summarized narratively and were not quantitatively pooled (Table 2).

**Table 2:** Summary of primary outcomes related to muscle health following Urolithin A supplementation in randomized controlled trials.

Domain	Outcome (units)	Study	Duration	UA arm: n, change (mean ± SD)	Placebo: n, change (mean ± SD)	Between-group MD (95% CI)	*p*-value
Strength	Quadriceps average peak torque (%)	Singh 2022	4 mo	29: +4.7 (SD NR)	30: −2.5 (SD NR)	+7.2 (95% CI NR)	>0.05
Strength	Hamstring average peak torque (%)	Singh 2022	4 mo	29: +9.8 (SD NR)	30: −9.8 (SD NR)	+19.6 (95% CI 2.0 to 37.2)	0.029
Strength	Hand-grip strength (%)	Singh 2022	4 mo	29: +5.1 (SD NR)	30: +2.4 (SD NR)	+2.7 (95% CI NR)	0.080
Strength	1RM bench press (kg)	Zhao 2024	8 wk	10: +3.0 ± 0.17	10: +0.5 (SD NR)	+3.5 (95% CI NR)	0.462
Strength	1RM back squat (kg)	Zhao 2024	8 wk	10: +1.35 ± 2.73	10: NR	2.55 (95% CI NR)	0.710
Strength	Quadriceps MVIC (Nm)	Zhao 2024	8 wk	10: +36.10 ± 0.62	10: NR	+43.5 (95% CI NR)	0.048
Strength	Maximal sprint speed (km/h)	Acevedo 2025	6 wk	10: 5.2 (SD NR)	10: 5.3 (SD NR)	NR	>0.05
Endurance/performance	FDI contraction time to fatigue (s)	Liu 2022	4 mo	30: +95.3 ± 115.5	30: +11.6 ± 147.4	+83.7 (95% CI NR)	<0.05
Endurance/performance	Tibialis anterior contraction time to fatigue (s)	Liu 2022	4 mo	30: +41.4 ± 65.5	30: +5.7 ± 127.1	+35.7 (95% CI NR)	NR
Endurance/performance	6-min walk distance (m)	Liu 2022	4 mo	30: +60.8 ± 67.2	30: +42.5 ± 73.3	+18.3 (95% CI NR)	NR
Endurance/performance	6-min walk distance (m)	Singh 2022 (500 + 1,000 mg combined)	4 mo	52: +15.69 ± 66.91	27: −0.52 ± 59.08	+16.21 (95% CI −12.56 to 44.97)	0.270
Endurance/performance	VO₂peak (%)	Singh 2022	4 mo	29: +10.2 (SD NR)	30: −1.1 (SD NR)	NR	0.058
Endurance/performance	Bench-press reps to failure (n)	Zhao 2024	8 wk	10: +2.00 ± 0.56	10: NR	+2.00 (95% CI NR)	0.011
Endurance/performance	Running economy / VO₂max change (%)	Whitfield 2025	4 wk	21: +5.4 ± 0.9	21: +3.6 ± 1.3	NR	0.138
Endurance/performance	Yo-Yo IR1 distance (m)	Acevedo 2025	6 wk	10: NR	10: NR	+239 (95% CI 20 to 454)	0.048
Endurance/performance	CMJ height (cm)	Acevedo 2025	6 wk	10: NR	10: NR	+3.3 (95% CI 0.88 to 5.95)	0.020
Endurance/performance	CMJ peak force (N)	Acevedo 2025	6 wk	10: NR	10: NR	−136 (95% CI −257 to −13)	0.046
Endurance/performance	MCJ peak power (W)	Acevedo 2025	6 wk	10: NR	10: NR	+25 (95% CI -162 to 215)	0.797
Mass	Lean body mass (DXA, %)	Singh 2022	4 mo	29: −1.0 (SD NR)	30: −0.7 (SD NR)	NR	NR
Biomarkers	CRP (mg/L)	Singh 2022	4 mo	25: NR	27: NR	NR	<0.05
Biomarkers	CRP (mg/L)	Liu 2022	4 mo	30: 2.07 ± 1.46	30: 2.65 ± 1.86	NR	NR
Biomarkers	CK tAUC	Whitfield 2025	4 wk	22: NR	20: NR	NR	0.891
Biomarkers	Free radical scavenging	Acevedo 2025	6 wk	10: NR	10: NR	NR	0.350
	Hydrogen Peroxide scavenging	Acevedo 2025	6 wk	10: NR	10: NR	NR	0.997
	Lipid/Organic Hydroperoxide scavenging	Acevedo 2025	6 wk	10: NR	10: NR	NR	0.353

Within-arm values are change from baseline unless otherwise indicated (mean ± SD). Where the source publication did not report a numerical mean or SD, the value is recorded as “NR” rather than narrative descriptors such as “induction,” “reduce,” or “significant reduction.” Between-group differences are mean differences (MD) UA minus placebo with 95% confidence intervals; *p*-values are two-sided and reported to three decimal places (or as “<0.001”/“<0.050”/“>0.050” when only a threshold was reported in the source publication).

1RM, one-repetition maximum; tAUC, total area under the curve; CMJ, countermovement jump; CK, creatine kinase; CRP, C-reactive protein; DXA, dual-energy X-ray absorptiometry; FDI, first dorsal interosseus; MVIC, maximal voluntary isometric contraction; NR, not reported; VO₂max, maximal oxygen consumption; VO₂peak, peak oxygen consumption; Yo-Yo IR1, Yo-Yo Intermittent Recovery Test Level 1.

Values mean changes from baseline unless otherwise indicated. (95% CI values are derived from reported where available).

Given the small number of trials, heterogeneous strength measures, and isolated significant findings, these strength results should be interpreted as exploratory, hypothesis-generating signals rather than as reproducible evidence of a strength benefit. (Table 2).

### 3.5 Muscle endurance and aerobic performance

Outcomes related to muscle endurance and aerobic performance were summarized narratively and were not quantitatively pooled. Individual trials reported improvements in contraction time to fatigue of the first dorsal interosseus and tibialis anterior in older adults [Liu et al., (9); *p* < 0.01 and *p* = 0.05, respectively], repetitions to failure during resistance exercise in trained male athletes (Zhao et al. (6); *p* = 0.011), and Yo-Yo Intermittent Recovery Test Level 1 distance and countermovement jump height in academy soccer players (Acevedo et al., (12); *p* < 0.05 and *p* = 0.02). VO₂peak in middle-aged adults [Singh et al., (5)] and running economy at altitude in highly trained distance runners [Whitfield et al., (11)] showed numerical improvements that did not reach statistical significance (*p* = 0.058 and *p* = 0.138, respectively). Lean body mass assessed by DXA was unchanged (Singh et al., (5), *p* > 0.05).

Taken together, these narrative findings are directionally consistent with a metabolic and fatigue-resistance effect of Urolithin A rather than structural hypertrophy. However, given the small number of trials, heterogeneity of outcome measures, and absence of quantitative pooling, the magnitude and generalizability of these effects cannot be reliably estimated from the current evidence base.

### 3.6 Functional capacity

**Figure 3:**
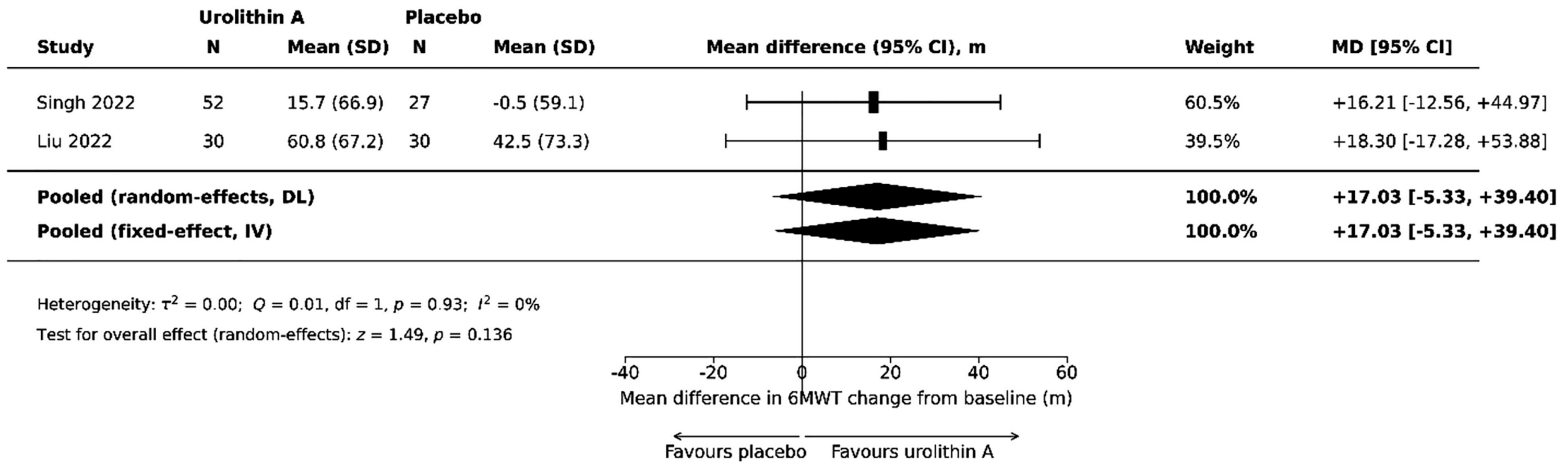
Effect of Urolithin A supplementation on functional exercise capacity assessed by the 6MWT. Forest plot of pooled mean differences in 6-min walk test (6MWT) distance following oral Urolithin A supplementation versus placebo. Effects are expressed as change-from-baseline (meters). The 500 mg and 1,000 mg Urolithin A arms of Singh et al. (2022) were combined into a single comparison against the shared placebo group following the Cochrane Handbook. The fixed-effect inverse-variance pooled mean difference is +17.03 m (95% CI − 5.33 to 39.40 m; *p* = 0.135; *I*^2^ = 0%); the random-effects (DerSimonian–Laird) estimate is identical (τ^2^ = 0).

Functional exercise capacity was evaluated using the 6MWT in two randomized controlled trials. Because Singh et al. (5) included two Urolithin A dose arms (500 mg and 1,000 mg) and a single shared placebo group, the two intervention arms were combined into a single comparison versus placebo following the Cochrane Handbook to avoid a unit-of-analysis error (see Methods). The pooled mean difference (UA minus placebo) was +17.03 m (95% CI −5.33 to 39.40 m; *Z* = 1.49; *p* = 0.135; *I*^2^ = 0%; Q = 0.008, *p* = 0.93; fixed-effect inverse-variance model). The random-effects DerSimonian–Laird estimate was identical (τ^2^ = 0). A sensitivity analysis using placebo-group splitting produced a comparable result (MD + 18.80 m; 95% CI −3.24 to 40.85 m; *p* = 0.095). Heterogeneity statistics for k = 2 are underpowered and are reported with caution (Figure 3). Compared with a preliminary analysis that included only the 1,000 mg arm of Singh et al. (5) (MD + 27.46 m, 95% CI 4.54–50.38 m, *p* = 0.019), correcting the unit-of-analysis error reduced the pooled estimate by approximately 38% and rendered the 6MWT difference no longer statistically significant.

Subgroup-level estimates were directionally consistent across age groups, with numerically larger improvements observed in middle-aged adults than in older adults, although confidence intervals overlapped and crossed the null effect. The certainty of evidence for the pooled 6MWT outcome was rated as low (GRADE) in Supplementary Table S4, reflecting the very small number of contributing trials, modest sample sizes, clinical heterogeneity across populations and intervention durations, and imprecision of the pooled estimate. These results should therefore be regarded as directionally favorable but statistically inconclusive, rather than as definitive evidence of a clinically meaningful effect on integrated cardiovascular–muscular performance.

### 3.7 Biochemical and mitochondrial biomarkers

Biochemical and mitochondrial biomarker findings were summarized narratively. Individual trials reported reductions in plasma acylcarnitines and ceramides [Liu et al., (9)], C-reactive protein [Singh et al., 2022 and Liu et al. (9)], and exercise-induced creatine kinase [Whitfield et al., (11)]. Some markers [e.g., salivary antioxidant capacity in Acevedo et al., (12)] decreased in both groups, complicating interpretation. Across the evidence base, biomarker assessments were not standardized between trials, sample sizes were small, and biomarker outcomes were not quantitatively pooled. These findings should therefore be regarded as preliminary and hypothesis-generating rather than as direct evidence of improved mitochondrial health.

## 4 Discussion

This systematic review synthesized evidence from five randomized controlled trials evaluating oral Urolithin A supplementation on muscle-related outcomes in adults and post-pubertal athletic populations. Quantitative synthesis was feasible only for the 6-min walk test (*k* = 2). After correcting a unit-of-analysis error in the previously reported pooled estimate — by combining the 500 mg and 1,000 mg Urolithin A arms of Singh et al. (5) against the shared placebo group following Cochrane Handbook guidance — the revised pooled mean difference for the 6MWT was +17.03 m (95% CI −5.33 to 39.40 m; *p* = 0.135), with low certainty of evidence (GRADE). All other outcomes, including muscle strength, endurance, aerobic capacity, lean mass, and biochemical or mitochondrial biomarkers, were summarized narratively. Individual trials reported isolated improvements in selected neuromuscular and biomarker outcomes, but these were not quantitatively pooled, used non-overlapping outcome panels, and arose from small, clinically heterogeneous studies; they should be treated as exploratory signals to be tested in adequately powered future trials, not as reproducible evidence of efficacy.

Age-related decline in mitochondrial quality control, particularly mitophagy, is a recognized feature of skeletal-muscle aging and sarcopenia (11–13). Mechanistic and preclinical work suggests that Urolithin A may engage this biology by activating PINK1/Parkin-mediated mitophagy and improving mitochondrial efficiency. The functional and biomarker signals reported in individual trials are biologically compatible with this mechanism but were not consistent across trials and were not pooled. However, the current human evidence base — limited to five small randomized controlled trials with heterogeneous populations and outcomes — does not yet allow us to conclude that Urolithin A meaningfully restores mitochondrial quality control in clinical settings, and we therefore frame the mechanistic discussion below as a hypothesis-generating context for the observed findings rather than as confirmed mechanistic translation.

The mechanistic rationale for Urolithin A in muscle health is supported by robust preclinical and translational evidence demonstrating that UA is a potent inducer of mitophagy through activation of the PINK1–Parkin signaling pathway (2). Experimental studies in *Caenorhabditis elegans*, rodent models, and human cell systems have consistently shown that UA enhances mitochondrial quality control, restores oxidative phosphorylation efficiency, and reduces reactive oxygen species accumulation (1). Human omics data further support these mechanisms, revealing enrichment of Parkin-mediated ubiquitin–proteasomal pathways, increased Parkin Ser65 phosphorylation, and dose-dependent upregulation of tricarboxylic acid cycle and oxidative phosphorylation proteins in skeletal muscle (5). Some of these molecular signatures parallel adaptations observed with endurance exercise training in preclinical models, but a direct translational link to the modest, non-pooled functional changes reported here has not been established.

Beyond the quantitatively pooled 6MWT outcome, the present synthesis provides narrative, non-pooled signals rather than quantitative corroboration of the mechanistic hypotheses. Individual trials reported improvements in endurance, maximal voluntary contraction, and fatigue resistance alongside reductions in circulating acylcarnitines, ceramides, C-reactive protein, and exercise-induced creatine kinase. We therefore consider these findings preliminary and hypothesis-generating, and they should not be interpreted as conclusive evidence that Urolithin A improves mitochondrial turnover or bioenergetic efficiency in humans.

With the revised analysis combining the Urolithin A arms of Singh et al. (5) against the shared placebo, the pooled 6MWT mean difference of +17.03 m (95% CI −5.33 to 39.40 m) is directionally favorable but statistically inconclusive (*p* = 0.135). This effect size lies below or near commonly cited minimal clinically important difference thresholds, which range from approximately 14 m in older adults and cardiopulmonary rehabilitation populations to 30–50 m in heart failure cohorts (14, 15). The clinical interpretation of this magnitude is therefore population-specific and should be made cautious. Findings from the academy soccer pilot [Acevedo et al., (12)] further suggest possible neuromuscular and aerobic signals under high training loads, but precision was limited and several outcomes shifted variably (12).

Apparent differences across the small set included populations older and middle-aged untrained adults, resistance-trained athletes, endurance runners, and academy soccer players — are descriptive rather than statistical: no formal subgroup or meta-regression analyses were feasible with only five clinically heterogeneous trials. These population-level patterns are exploratory hypotheses for future trials, not established moderators of response.

Strengths of this review include the prospective PROSPERO registration, PRISMA 2020-compliant reporting, use of an *a priori* PICOS framework, exclusive inclusion of randomized controlled trials, independent duplicate screening and data extraction, risk-of-bias assessment using Cochrane RoB 2, GRADE certainty-of-evidence assessment for the primary pooled outcome, and explicit handling of the multi-arm Singh trial with both pooled-arm and placebo-splitting sensitivity analyses.

Several important limitations constrain interpretation. First, quantitative pooling was feasible for only one outcome (6MWT) and was based on only two trials, so the pooled estimate is imprecise and was rated as low certainty by GRADE; the remaining muscle, performance, and biomarker outcomes are reported narratively and remain exploratory. Second, the overall evidence base comprises only five small randomized controlled trials with modest sample sizes, short intervention durations (4 weeks to 4 months), and substantial clinical heterogeneity in age, training status, sex distribution, and outcome measurement; the included populations span older adults, middle-aged untrained adults, resistance-trained male athletes, highly trained male endurance runners, and post-pubertal academy soccer players, which limits clinical interpretability. One trial enrolled post-pubertal academy soccer players (mean age ~17.5 years), which broadens the represented population beyond strictly adult cohorts and limits generalization to aging-related functional decline. Third, biomarker assessments and outcome definitions were not harmonized across trials, and the small number of studies precluded formal evaluation of publication bias and dose–response relationships. Fourth, we did not pre-register specific subgroup analyses; subgroup patterns described in the Results and Discussion are therefore exploratory. Fifth, intervention durations were short (4 weeks to 4 months), so durability of any observed effects and long-term safety cannot be inferred. Finally, all included trials were published in English; non-English trials, unpublished data, and ongoing studies could affect future updates of this synthesis.

In conclusion, the current randomized human evidence base for Urolithin A supplementation on muscle-related outcomes remains limited and short-term. The corrected meta-analysis of the 6MWT (k = 2) shows a directionally favorable but statistically inconclusive effect with low GRADE certainty, and the remaining muscle, performance, and biomarker outcomes are reported narratively as exploratory, hypothesis-generating signals. Larger, longer-duration, methodologically standardized randomized controlled trials in better-defined and more homogeneous populations are required before clinical recommendations regarding Urolithin A for muscle health, including for aging-related functional decline, can be made.

## Data Availability

The original contributions presented in the study are included in the article/Supplementary material, further inquiries can be directed to the corresponding author.
