# Supplementary material for: Effects of Urolithin A supplementation on muscle health outcomes in humans from randomized controlled trials
Source: Front Nutr. 2026 Jul 22;13:1834344. doi: 10.3389/fnut.2026.1834344 (PMC13440176; doi:10.3389/fnut.2026.1834344)
Supplement: Supplementary file 1 [file Supplementary_file_1.docx]

**Supplementary Table S1:** Boolean search **strings used in each database**

| **Database** | **Final search date** | **Field tags / scope** | **Full Boolean search string** |
| --- | --- | --- | --- |
| PubMed | 10 Nov 2025 | Default (All Fields) | ( ( ( "urolithin a" OR "urolithin" OR "urolithins" ) AND ( "randomized controlled trial" OR "randomised controlled trial" OR "controlled clinical trial" OR "placebo" OR "intervention" OR "trial" OR "experimental study" ) AND ( "muscle" OR "skeletal muscle" OR "sarcopenia" OR "muscle strength" OR "physical performance" OR "mitochondria" OR "mitochondrial function" OR "muscle mass" OR "lean mass" OR "frailty" OR "muscle protein" OR "walking speed" OR "gait speed" OR "handgrip strength" OR "grip strength" OR "lean body mass" OR "fat free mass" ) ) ) |
| Embase | 15 Dec 2025 | Default (multi-field) | ( "urolithin a" OR "urolithin" OR "urolithins" ) AND ( "randomized controlled trial" OR "randomised controlled trial" OR "controlled clinical trial" OR "placebo" OR "intervention" OR "trial" OR "experimental study" ) AND ( "muscle" OR "skeletal muscle" OR "sarcopenia" OR "muscle strength" OR "physical performance" OR "mitochondria" OR "mitochondrial function" OR "muscle mass" OR "lean mass" OR "frailty" OR "muscle protein" OR "walking speed" OR "gait speed" OR "handgrip strength" OR "grip strength" OR "lean body mass" OR "fat free mass" ) |
| Web of Science (Core Collection: SCI-EXPANDED, CPCI-S, CPCI-SSH) | 10 Nov 2025 | Smart Search (Topic); Document Types = Article OR Early Access OR Proceeding Paper OR Editorial Material (70 records returned) | ( ( "urolithin a" OR "urolithin" OR "urolithins" ) AND ( "randomized controlled trial" OR "randomised controlled trial" OR "controlled clinical trial" OR "placebo" OR "intervention" OR "trial" OR "experimental study" ) AND ( "muscle" OR "skeletal muscle" OR "sarcopenia" OR "muscle strength" OR "physical performance" OR "mitochondria" OR "mitochondrial function" OR "muscle mass" OR "lean mass" OR "frailty" OR "muscle protein" OR "walking speed" OR "gait speed" OR "handgrip strength" OR "grip strength" OR "lean body mass" OR "fat free mass" ) ) AND DT=(Article OR Early Access OR Proceeding Paper OR Editorial Material) |
| Scopus | 10 Nov 2025 | TITLE-ABS-KEY | TITLE-ABS-KEY ( ( ( "urolithin a" OR "urolithin" OR "urolithins" ) AND ( "randomized controlled trial" OR "randomised controlled trial" OR "controlled clinical trial" OR "placebo" OR "intervention" OR "trial" OR "experimental study" ) AND ( "muscle" OR "skeletal muscle" OR "sarcopenia" OR "muscle strength" OR "physical performance" OR "mitochondria" OR "mitochondrial function" OR "muscle mass" OR "lean mass" OR "frailty" OR "muscle protein" OR "walking speed" OR "gait speed" OR "handgrip strength" OR "grip strength" OR "lean body mass" OR "fat free mass" ) ) ) |

**Supplementary Table S2:** PICOS framework defining the eligibility criteria for this systematic review and meta-analysis.

| **PICOS Element** | **Eligibility Criteria** |
| --- | --- |
| Population (P) | Adults and post-pubertal/late-adolescent athletic populations evaluated for muscle function, physical performance, or mitochondrial-related outcomes. |
| Intervention (I) | Oral Urolithin A supplementation at any dose or duration. |
| Comparator (C) | Placebo or no supplementation. |
| Outcomes (O) | Muscle strength, muscle mass, physical performance (including 6-minute walk test [6MWT]), and mitochondrial or metabolic biomarkers. Studies were required to report extractable quantitative outcome data for at least one eligible endpoint. |
| Study Design (S) | Randomized controlled trials (RCTs) involving human participants and published in English. Non-randomized studies, non-human studies, review articles, conference abstracts, editorials, and studies lacking extractable data were excluded. |

**Supplementary Table S3:** Baseline participant characteristics of included randomized controlled trials

| **Variable** | **Singh et al., 2022 (*Cell Rep Med*)** | **Liu et al., 2022 (*JAMA Netw Open*)** | **Zhao et al., 2024 (*JISSN*)** | **Whitfield et al., 2025 (*Sports Med*)** | **Acevedo et al., 2025 (Front Nutr)** |
| --- | --- | --- | --- | --- | --- |
| Population (n) | 88 (29 placebo; 29 UA 500 mg; 30 UA 1000 mg) | 66 (33 placebo; 33 UA 1000 mg) | 20 (10 placebo; 10 UA 1000 mg) | 42 (21 placebo; 21 UA 1000 mg) | 20 (n = 10 UA; n = 10 placebo) |
| Age (years, mean ± SD) | 54.38 ± 6.42 (placebo); 51.03 ± 7.16 (500 mg); 52.07 ± 5.56 (1000 mg) | 71 ± 4.58 (placebo); 72 ± 5.24 (UA) | 24.1 ± 1.6 (UA baseline) | 27.2 ± 3.5 (UA baseline) | 17.2 ± 3.5 (1000mg), 17.8 ± 3.8 (placebo) |
| Sex (% male) | 31 (placebo); 38 (500 mg); 40 (1000 mg) | 30 (placebo); 18 (UA) | 100 (male athletes) | 100 (male runners) | 100 (male soccer players) |
| BMI (kg/m²) | 29.3 ± 2.55 (placebo); 29.52 ± 2.82 (500 mg); 28.91 ± 2.89 (1000 mg) | 26.7 ± 3.77 (placebo); 25.7 ± 3.45 (UA) | – | – |  |
| Systolic BP (mmHg) | 123.7 ± 11.9 (placebo); 124.8 ± 11.4 (500 mg); 125.5 ± 10.2 (1000 mg) | 132.1 ± 13.0 (placebo); 132.9 ± 13.6 (UA) | – | – |  |
| Diastolic BP (mmHg) | 79.0 ± 8.7 (placebo); 78.4 ± 6.9 (500 mg); 80.1 ± 7.3 (1000 mg) | 75.5 ± 8.0 (placebo); 76.8 ± 9.2 (UA) | – | – |  |
| Heart rate (bpm) | 65.7 ± 9.6 (placebo); 67.6 ± 7.2 (500 mg); 67.9 ± 8.6 (1000 mg) | 64.4 ± 9.9 (placebo); 65.4 ± 9.6 (UA) | – | – |  |

**Supplementary Table S4:** GRADE Summary of Findings for 6-minute walk test (6MWT)

| **Outcome** | **Participants (studies)** | **Relative effect / pooled estimate** | **Certainty of the evidence (GRADE)** | **Comments** |
| --- | --- | --- | --- | --- |
| 6-minute walk test (6MWT), change from baseline | 139 participants (2 RCTs) | MD +17.03 m (95% CI [1] −5.33 to 39.40); fixed-effect inverse-variance model; I² = 0%, Q = 0.008, p = 0.93 | Low ᵃᵇᶜᵈᵉ | Direction of effect favored urolithin A, but the confidence interval crossed both no effect and a clinically important benefit threshold; result not statistically significant (p = 0.135). |

ᵃ Studies included. Liu et al. (2022) and Singh et al. (2022).

ᵇ Singh et al. (2022) included two urolithin A intervention arms sharing a common placebo group. The intervention arms were combined before meta-analysis according to Cochrane guidance to avoid a unit-of-analysis error. Sensitivity analyses using placebo-group splitting produced similar results.

ᶜ Risk of bias. Downgraded one level for risk of bias. Liu et al. (2022) changed the primary endpoint during the trial, and Singh et al. (2022) had imbalanced attrition between groups. In both studies, assessor blinding for 6MWT was not clearly reported, and both trials were industry-sponsored.

ᵈ Indirectness. Downgraded one level for indirectness because both trials enrolled generally healthy or overweight older adults rather than clearly defined clinical populations with impaired functional capacity, which may limit direct applicability to broader patient populations.

ᵉ Imprecision. Downgraded one level for imprecision. The total sample size was modest (n = 139), and the pooled 95% confidence interval (-5.33 to 39.40 m) crossed both the null and an approximate minimal clinically important difference for 6MWT of about 30 m. The corrected pooled estimate was not statistically significant.
